# Efficacy and Safety of Antibiotics for Treatment of Scrub Typhus: A Network Meta-analysis

**DOI:** 10.1001/jamanetworkopen.2020.14487

**Published:** 2020-08-28

**Authors:** Jiaru Yang, Lisha Luo, Taigui Chen, Lianbao Li, Xin Xu, Yu Zhang, Wenjing Cao, Peng Yue, Fukai Bao, Aihua Liu

**Affiliations:** 1 Institute for Tropical Medicine, Kunming Medical University School of Basic Medical Sciences, Kunming, China; 2 Yunnan Province Key Laboratory for Major Children Diseases Research, The Childrens Hospital of Kunming, Kunming Medical University, Kunming, China; 3 Yunnan Province Key Laboratory for Tropical Infectious Diseases in Universities, Kunming Medical University, Kunming, China

## Abstract

**Importance:**

Antibiotics have been used for many years to treat scrub typhus, but their efficacy and safety have not been studied thoroughly.

**Objective:**

To compare and rank different antibiotics to identify which one can safely eliminate *Orientia tsutsugamushi* and efficiently alleviate fever in patients with scrub typhus.

**Data Sources:**

An electronic search of PubMed and Embase was conducted, from database inception to July 12, 2019. The study was conducted from July 12 to September 2, 2019.

**Study Selection:**

Randomized clinical trials and retrospective studies that evaluated the use of antibiotics for treatment in patients diagnosed with scrub typhus caused by *O tsutsugamushi* were included. Records of articles in English were considered eligible. Studies were assessed independently by 2 reviewers, with disagreement resolved by consensus. Of 6408 studies initially identified, 10 randomized clinical trials and 4 retrospective study met the criteria for further analysis.

**Data Extraction and Synthesis:**

This study followed the Preferred Reporting Items for Systematic Reviews and Meta-analyses extension statement for systematic reviews incorporating network meta-analyses of health care interventions. Data were independently extracted by 2 reviewers and synthesized with frequentist random-effects network meta-analyses.

**Main Outcomes and Measures:**

The primary outcome was efficacy of the antibiotic, considered as the number of patients who achieved complete healing with an antibiotic. Safety, defined as the prevalence of adverse events associated with the antibiotics, was the secondary outcome, and defervescence time was the tertiary outcome. P scores (scale of 0 to 1, with 1 indicating superiority to other treatments) were used to rank the efficacy, safety, and defeverescence time of the antibiotics.

**Results:**

Three searches for articles in Embase and PubMed identified 10 randomized clinical trials (888 participants) and 4 retrospective studies (323 participants) for further analyses. No particular treatment regimen showed a significant advantage or disadvantage with regard to efficacy or safety. However, meta-analysis of retrospective studies indicated that clarithromycin (P score = 0.8730) alleviated fever more efficiently than other antibiotics.

**Conclusions and Relevance:**

No treatment regimen reported in this network meta-analysis showed a significant advantage or disadvantage with regard to efficacy or safety. However, clarithromycin might be a better choice than the other drugs for alleviating fever.

## Key Points

**Question:**

Which antibiotic is associated with the greatest efficacy and safety for treating scrub typhus?

**Findings:**

In this network meta-analysis of 14 studies, 8 antibiotics that are the most commonly used for treating scrub typhus showed no significant advantage or disadvantage with regard to efficacy or safety. In retrospective studies, clarithromycin alleviated fever more efficiently than other antibiotics.

**Meaning:**

The findings of analysis for efficacy, safety, and defervescence time of these 8 antibiotics may provide a reference for clinical decision-making.

## Teaser

This network meta-analysis compares the efficacy and safety of 8 antibiotics used for treatment of scrub typhus.

## Introduction

*Orientia tsutsugamushi* is a type of gram-negative, obligately intracellular bacillus that belongs to the order Rickettsiales within the family Rickettsiaceae.^1^ Infection by *O tsutsugamushi* can lead to scrub typhus in humans.^2^

Scrub typhus has wide distribution in tropical and subtropical regions, such as the Arabian Peninsula, Chile, and possibly Kenya.^3^ Scrub typhus is transmitted via arthropods and, currently, 1 billion people are potentially exposed to scrub typhus worldwide.^4^ In Southeast Asia, scrub typhus is a leading cause of treatable febrile disease besides malaria. However, although almost 1 million new cases are reported every year, scrub typhus is regarded as a neglected tropical disease.^5^

The clinical manifestations of scrub typhus differ among individuals. Almost 5 to 14 days after being bitten by *Leptotrombidium* mites, patients exhibit rash and eschar at the bite site, as well as fever, headache, myalgia, cough, generalized lymphadenopathy, nausea, vomiting, and abdominal pain. Of these, fever and headache are the most frequently reported manifestations among patients with scrub typhus^4,6,7,8^; some studies have reported that more than 95% of patients with confirmed cases of scrub typhus have fever.^9,10^ In some individuals, scrub typhus can lead to multiorgan dysfunction, with possible mortality rates of 30%.^11^

The World Health Organization has declared scrub typhus to be one of the most underdiagnosed/underreported diseases worldwide that often necessitates hospitalization.^1^ If patients do not receive sufficiently early and effective treatment, scrub typhus may induce interstitial pneumonia, acute respiratory distress syndrome, meningoencephalitis, acute kidney injury, or disseminated intravascular coagulation, which causes death in 7% of patients with the infection.^12^ Hence, a thorough understanding of the therapy of scrub typhus is important.

Antibiotics have been used for scrub typhus treatment for many years and, with no vaccine available, are the only way to treat scrub typhus.^5^ Thus, it is necessary to assess the antibiotics used in treatment. The most common antibiotics used for treatment are doxycycline, tetracyclines, chloramphenicol, and azithromycin,^13^ but their efficacy is disputable. Although studies have compared the efficacy of some antibiotics for curing scrub typhus,^13,14,15,16,17^ these studies have not been comprehensive or quantitative. This lack of understanding the varying efficacy of the drugs may lead to patients getting sicker because of inappropriate treatment regimens.

Thus, we used a network meta-analysis to systematically analyze data derived from randomized clinical trials (RCTs) and retrospective studies to evaluate the use of various antibiotics against scrub typhus. In this way, we hope to provide evidence for clinicians to develop therapeutic schedules.

## Methods

This network meta-analysis was undertaken on the basis of the Preferred Reporting Items for Systematic Reviews and Meta-Analyses (PRISMA) extension statement for systematic reviews incorporating network meta-analyses of health care interventions.^18^ Using the Grading of Recommendations Assessment, Development and Evaluation (GRADE) system,^19,20,21,22,23,24^ we assessed the certainty of evidence derived from network meta-analysis results. GRADE provides a system for rating the quality of systematic reviews, meta-analyses, or network meta-analyses.^25^ The GRADE system evaluates the quality of evidence at 4 levels: high, moderate, low, and very low.

### Search Strategies and Inclusion Criteria

The study was conducted from July 12 to September 2, 2019. We searched articles in Embase and PubMed databases from the date of their inception to July 12, 2019, on 3 occasions. In the first search, we used *scrub typhus* combined with a list of antibiotics. This list included 11 antibiotics: *chloramphenicol*, *tetracycline*, *doxycycline*, *rifampicin*, *erythromycin*, *azithromycin*, *telithromycin*, *levofloxacin*, *minocycline*, *penicillin*, and *aureomycin*. For the second search, the search terms were *scrub typhus*, *therapy*, *treatment*, *cure*, *drug*, *antibiotic*, and *antimicrobial*. For the final search, we used *scrub typhus*, *randomized controlled trial*, *controlled clinical trial*, *random allocation*, *double-blind*, *single-blind*, *survival*, *treatment*, *therapy*, *comparison*, *comparative*, *effective*, and *efficacy* as search terms.

All studies had to be RCTs or retrospective studies that compared the efficacy or safety of drugs used to treat scrub typhus and published in English. In addition, all patients in RCTs or retrospective studies had to have been diagnosed by clinicians in accordance with clinical symptoms and the results of laboratory tests to ascertain whether *O tsutsugamushi* was present in their body. Clinicians had to diagnose the disease in those patients using the Weil-Felix test, immunofluorescence assay, bacterial culture, enzyme-linked immunosorbent assay, or polymerase chain reaction test.

All included studies were assessed independently by 2 of us (L. Luo and T.C.). Disagreement for a particular assessment was resolved by discussing the issues until a consensus was reached.

### Data Extraction and Outcomes

We extracted data on patients and interventions from each study included in the network meta-analysis. For patient data, we recorded their age and sex. Furthermore, we noted the total number of patients, the number of patients treated using a particular antibiotic, and the number of patients who responded to a particular drug (complete recovery from scrub typhus).

Fever is the most commonly reported clinical manifestation of scrub typhus and is a strong indicator for evaluating therapeutic effects. Therefore, we recorded the defervescence time (mean [SD]) of patients treated with a particular drug. The unit of defervescence time is hours. For studies that did not specify the mean (SD) defervescence time, we extracted the median (range) values of the defervescence time and then calculated the mean (SD) using the method of McGrath and colleagues.^26^ To evaluate drug safety, we recorded the number of patients who developed an adverse reaction after receiving antibiotics. For data on interventions, we recorded the drug name, dose, and duration of therapy.

We evaluated all drugs systematically by 3 outcomes: efficacy, safety, and the defervescence time. Efficacy was the primary study outcome and referred to the response of patients to a particular drug and was measured by the total number of patients who recovered completely. The secondary outcome, safety, referred to the prevalence of adverse reactions of a particular drug and was measured by the total number of patients who developed an adverse reaction during or after treatment. The third outcome, defervescence time, was the time needed for abatement of fever, as indicated by a decrease in body temperature, after use of antibiotics. All data were extracted by 2 of us (L. Li and X.X.) independently.

### Statistical Analysis

Before we started to analyze extracted data, we assessed the risk of bias of all included studies in accordance with the tool for assessing risk of bias in randomized trials published by the Cochrane Collaboration.^27^ A network meta-analysis provides a generalization of pairwise meta-analysis that compares all pairs of interventions within several treatments for the same condition.^28,29^ We evaluated the efficacy, safety, and defervescence time of various antibiotics using a network meta-analysis.^30,31^ Furthermore, a random effects model and consistency model were used for analyzing data and carrying out the network meta-analysis. Odds ratios (ORs) were used to report the effect size for assessing efficacy and safety. Mean deviations (MDs) were used to report the effect size for assessing the time of defervescence.

Inconsistency (the difference of estimates of effect between direct evidence and indirect evidence) is an important indicator for a network meta-analysis. We used the back calculation method to assess the inconsistency of this network meta-analysis, which is based on the *Z* test, and provide the *P* value to define the inconsistency. That is, *P* < .05 denotes inconsistency in a network meta-analysis.^32^

To rank the efficacy, safety, and defervescence time of antibiotics, we used the P score as an indicator. The P score is used to measure the extent of certainty that a treatment is better than other treatments, averaged over all competing treatments.^33,34^ The P score is measured on a scale from 0 (worst) to 1 (best). Hence, if one treatment is better than the other treatments, its P score will be larger.

All analyses were conducted using the Netmeta package of R, version 3.5.2 (R Foundation) Stata, version 14.0 (StataCorp) (eTable 1 in the Supplement).

## Results

### Study Characteristics

**Figure 1.**
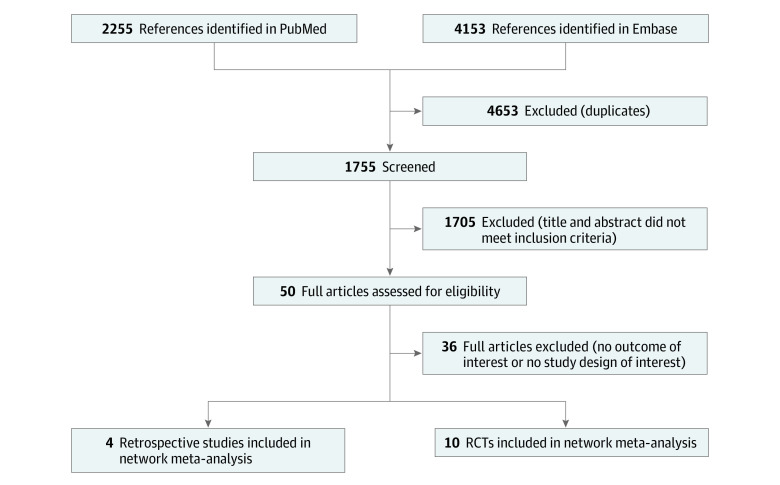
PRISMA Flow Diagram PRISMA indicates Preferred Reporting Items for Systematic Reviews and Meta-analyses; RCT, randomized clinical trial.

Our 3 searches of Embase and PubMed databases yielded 6408 articles (2255 from PubMed and 4153 from Embase). By excluding duplicate and ineligible studies, we selected 10 RCTs^35,36,37,38,39,40,41,42,43,44^ and 4 retrospective studies^45,46,47,48^ for further analyses (Figure 1).

The selected studies were published from 1973 to 2018 and involved 1211 patients (323 patients in retrospective studies and 888 patients in RCTs). In the 10 RCTs, 9 compared drug efficacy,^35,36,37,38,40,41,42,43,44^ 8 compared the defervescence time,^35,38,39,40,41,42,43,44^ and 8 compared adverse reactions^36,37,38,39,40,41,43,44^ (eTable 2, eTable 3, and eTable 4 in the Supplement). All 4 retrospective studies compared the efficacy of 5 drugs, and 3 of them^45,47,48^ compared the defervescence time (eTable 3 and 4 in the Supplement).

**Figure 2.**
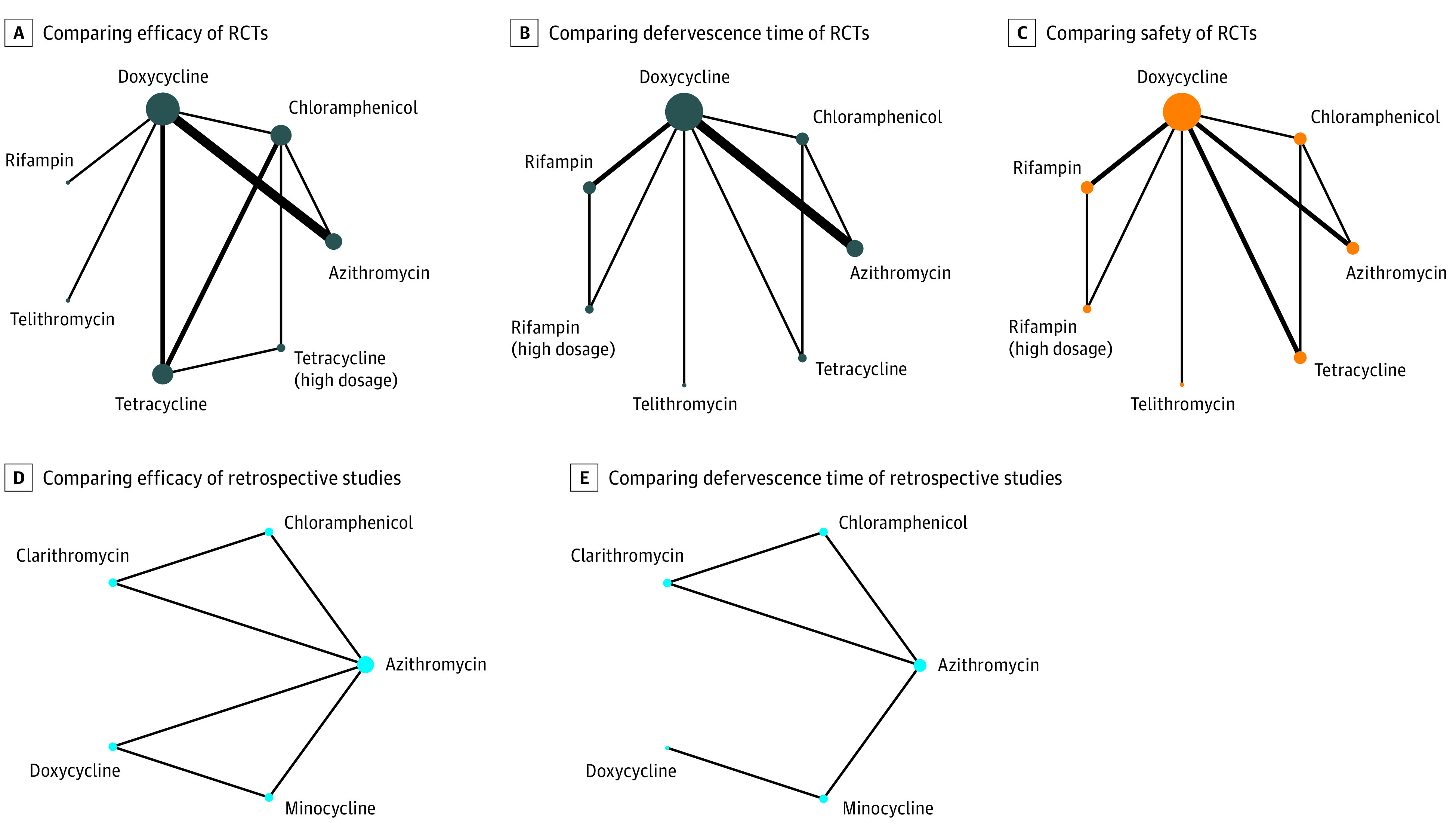
Network Meta-analysis Graphs of Antibiotics in Randomized Clinical Trials (RCTs) and Retrospective Studies Line width is proportional to the number of studies comparing every pair of treatments. Size of every circle is proportional to the number of patients. A, network meta-analysis graph of drugs for comparing efficacy of RCTs. B, network meta-analysis graph of drugs for comparing the defervescence time of RCTs. C, network meta-analysis graph of drugs for comparing safety of RCTs. D, network meta-analysis graph of drugs for comparing efficacy of retrospective studies. E, network meta-analysis graph of drugs for comparing the defervescence time of retrospective studies.

Evaluation of a bias risk for all RCTs is presented in eFigure 1 in the Supplement. The tool for assessing risk of bias in randomized trials examined RCTs according to 7 standards. Most of the included RCTs met most of these 7 standards, which indicated that the selected RCTs were of good quality. Network meta-analysis graphs developed for comparison of efficacy, defervescence time, and safety of the RCTs (Figure 2A, B, and C) and retrospective studies (Figure 2D and E) are provided.

### Outcomes

**Figure 3.**
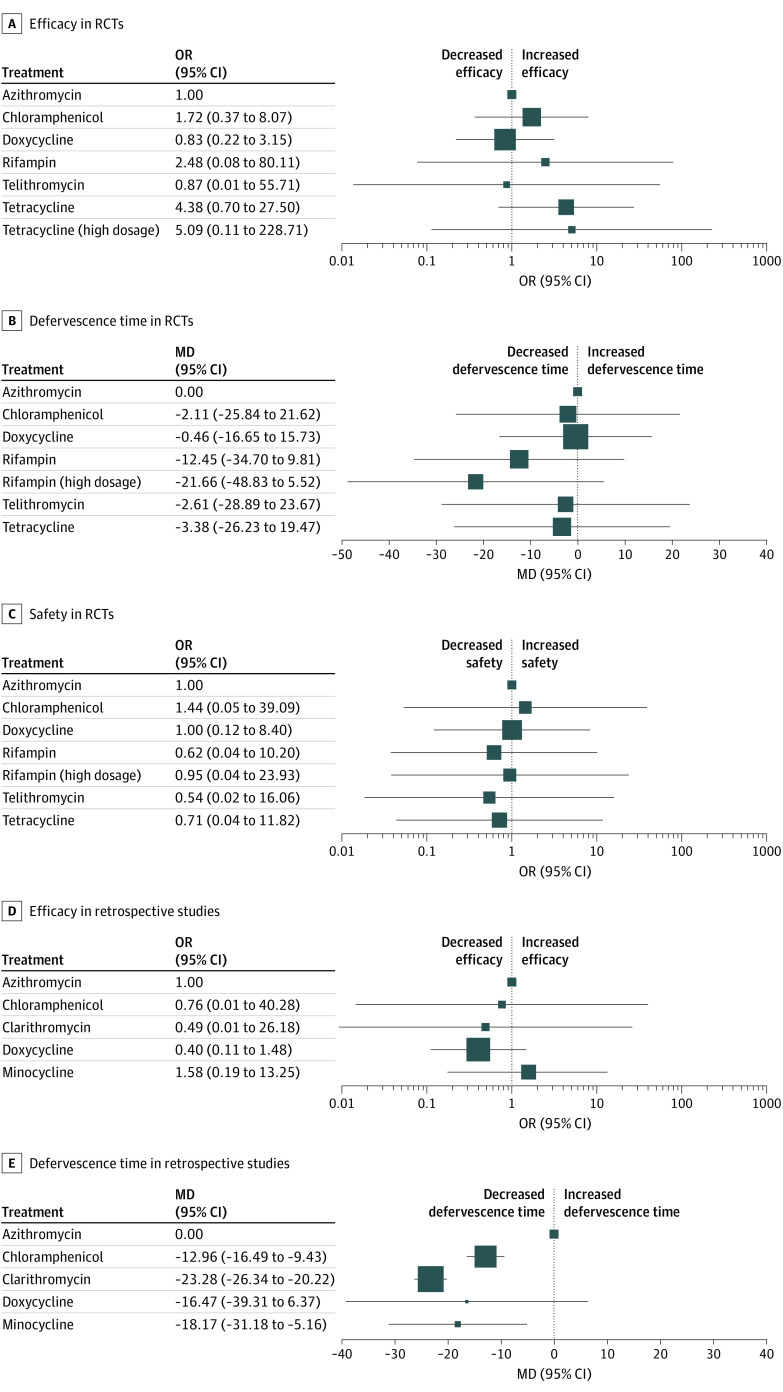
Network Meta-analysis for All Studies and Ranking of the Defervescence Time of Antibiotics Antibiotics vs azithromycin (reference drug). A, comparisons of drugs in RCTs for efficacy. B, comparisons of drugs in RCTs for the defervescence time. C, comparisons of drugs in RCTs for safety. D, comparisons of drugs in retrospective studies for efficacy. E, comparisons of drugs in retrospective studies for the defervescence time. MD indicates mean deviation; OR, odds ratio.

By analyzing data from the RCTs, we assessed the efficacy of 7 treatment regimens (Figure 2A). For retrospective studies, we assessed the efficacy of 5 drugs (Figure 2D). Comparison of results is presented using ORs (95% CIs). We compared the efficacy of all treatment regimens with that of azithromycin (reference drug), but a significant difference among them was not observed (Figure 3A and D).

Next, we conducted pairwise comparisons of the efficacy of all treatment regimens in RCTs (eFigure 2 in the Supplement) and retrospective studies (Table). There was no significant difference in efficacy among those treatments.

**Table.** Pairwise Comparisons of Drugs in RCTs and Retrospective Studies for Defervescence Time and Efficacy^a^

Comparison	Direct evidence				Indirect evidence			Network meta-analysis			*P* Value for test of inconsistency^**b**^
	No. of comparisons	MD (95% CI)	OR (95% CI)	Certainty of evidence	MD (95% CI)	OR (95% CI)	Certainty of evidence	MD (95% CI)	OR (95% CI)	Certainty of evidence	
Azithromycin vs chloramphenicol	1	14.98 (−18.05 to 48.0)	NA	Low^c^^,^^d^	−11.62 (−45.75 to 22.50)	NA	Low^c^^,^^d^	2.11 (−21.62 to 25.84)	NA	Low^c^^,^^d^	.27
Azithromycin vs doxycycline	3	−2.20 (−19.06 to 14.6)	NA	Low^c^^,^^d^	31.59 (−26.15 to 89.33)	NA		0.46 (−15.73 to 16.65)	NA	Low^c^^,^^d^	.27
Azithromycin vs rifampin	0	NA	NA	NA	12.45 (−9.81 to 34.70)	NA	Low^c^^,^^d^	12.45 (−9.81 to 34.70)	NA	Low^c^^,^^d^	NA
Azithromycin vs rifampin (high dosage)	0	NA	NA	NA	21.66 (−5.52 to 48.83)	NA	Low^c^^,^^d^	21.66 (−5.52 to 48.83)	NA	Low^c^^,^^d^	NA
Azithromycin vs telithromycin	0	NA	NA	NA	2.61 (−23.67 to 28.89)	NA	Low^c^^,^^d^	2.61 (−23.67 to 28.89)	NA	Low^c^^,^^d^	NA
Azithromycin vs tetracycline	0	NA	NA	NA	3.38 (−19.47 to 26.23)	NA	Low^c^^,^^d^	3.38 (−19.47 to 26.23)	NA	Low^c^^,^^d^	NA
Chloramphenicol vs doxycycline	1	−9.44 (−57.86 to 38.9)	NA	Low^c^^,^^d^	0.44 (−24.63 to 25.51)	NA	Low^c^^,^^d^	−1.65 (−23.91 to 20.61)	NA	Low^c^^,^^d^	.72
Chloramphenicol vs rifampin	0	NA	NA	NA	10.34 (−16.66 to 37.34)	NA	Low^c^^,^^d^	10.34 (−16.66 to 37.34)	NA	Low^c^^,^^d^	NA
Chloramphenicol vs rifampin (high dosage)	0	NA	NA	NA	19.55 (−11.63 to 50.72)	NA	Low^c^^,^^d^	19.55 (−11.63 to 50.72)	NA	Low^c^^,^^d^	NA
Chloramphenicol vs telithromycin	0	NA	NA	NA	0.50 (−29.90 to 30.90)	NA	Low^c^^,^^d^	0.50 (−29.90 to 30.90)	NA	Low^c^^,^^d^	NA
Chloramphenicol vs tetracycline	1	6.90 (−14.26 to 28.0)	NA	Moderate^d^	−18.18 (−57.52 to 21.15)	NA	Moderate^d^	1.27 (−17.36 to 19.09)	NA	Moderate^d^	.27
Doxycycline vs rifampin	2	11.99 (−3.28 to 27.26)	NA	Low^c^^,^^d^	NA	NA	NA	11.99 (−3.28 to 27.26)	NA	Low^c^^,^^d^	NA
Doxycycline vs rifampin (high dosage)	1	26.46 (2.57-50.35)	NA	Low^c^^,^^d^	−5.36 (−59.02 to 48.29)	NA	Low^c^^,^^d^	21.20 (−0.63 to 43.02)	NA	Low^c^^,^^d^	.29
Doxycycline vs telithromycin	1	2.15 (−18.55 to 22.8)	NA	Low^c^^,^^d^	NA	NA	NA	2.15 (−18.55 to 22.85)	NA	Low^c^^,^^d^	NA
Doxycycline vs tetracycline	1	−3.00 (−24.70 to 18.7)	NA	Low^c^^,^^d^	22.08 (−16.95 to 61.12)	NA	Low^c^^,^^d^	2.92 (−16.05 to 21.89)	NA	Low^c^^,^^d^	.27
Rifampin vs rifampin (high dosage)	1	4.49 (−18.73 to 27.7)	NA	Low^c^^,^^d^	38.05 (−19.37 to 95.48)	NA	Low^c^^,^^d^	9.21 (−12.32 to 30.73)	NA	Low^c^^,^^d^	.29
Rifampin vs telithromycin	0	NA	NA	NA	−9.84 (−35.56 to 15.88)	NA	Low^c^^,^^d^	−9.84 (−35.56 to 15.88)	NA	Low^c^^,^^d^	NA
Rifampin vs tetracycline	0	NA	NA	NA	−9.07 (−33.42 to 15.28)	NA	Low^c^^,^^d^	−9.07 (−33.42 to 15.28)	NA	Low^c^^,^^d^	NA
Rifampin (high dosage) vs telithromycin	0	NA	NA	NA	−19.05 (−49.12 to 11.03)	NA	Low^c^^,^^d^	−19.05 (−49.12 to 11.03)	NA	Low^c^^,^^d^	NA
Rifampin (high dosage) vs tetracycline	0	NA	NA	NA	−18.27 (−47.19 to 10.64)	NA	Low^c^^,^^d^	−18.27 (−47.19 to 10.64)	NA	Low^c^^,^^d^	NA
Telithromycin vs tetracycline	0	NA	NA	NA	0.77 (−27.30 to 28.85)	NA	Low^c^^,^^d^	0.77 (−27.30 to 28.85)	NA	Low^c^^,^^d^	NA
Azithromycin vs chloramphenicol	1	12.96 (9.43-16.49)	NA	NA	NA	NA	NA	12.96 (9.43-16.49)^e^	NA	NA	NA
Azithromycin vs clarithromycin	1	23.28 (20.22-26.34)	NA	NA	NA	NA	NA	23.28 (20.22-26.34)^e^	NA	NA	NA
Azithromycin vs doxycycline	0	NA	NA	NA	16.47 (−6.37 to 39.31)	NA	NA	16.47 (−6.37 to 39.31)	NA	NA	NA
Azithromycin vs minocycline	1	18.17 (5.16-31.18)	NA	NA	NA	NA	NA	18.17 (5.16-31.18)^e^	NA	NA	NA
Chloramphenicol vs clarithromycin	1	10.32 (6.09-14.55)	NA	NA	NA	NA	NA	10.32 (6.09-14.55)^e^	NA	NA	NA
Chloramphenicol vs doxycycline	0	NA	NA	NA	3.51 (−19.60 to 26.62)	NA	NA	3.51 (−19.60 to 26.62)	NA	NA	NA
Chloramphenicol vs minocycline	0	NA	NA	NA	−4.96 (−16.00 to 6.08)	NA	NA	−4.96 (−16.00 to 6.08)	NA	NA	NA
Clarithromycin vs doxycycline	0	NA	NA	NA	5.21 (−8.27 to 18.69)	NA	NA	5.21 (−8.27 to 18.69)	NA	NA	NA
Clarithromycin vs minocycline	0	NA	NA	NA	−5.11 (−18.47 to 8.25)	NA	NA	−5.11 (−18.47 to 8.25)	NA	NA	NA
Doxycycline vs minocycline	1	1.70 (−17.07 to 20.4)	NA	NA	NA	NA	NA	1.70 (−17.07 to 20.47)	NA	NA	NA
Azithromycin vs chloramphenicol	1	NA	1.308 (0.025-68.875)	NA	NA	NA	NA	NA	1.308 (0.025-68.875)	NA	NA
Azithromycin vs clarithromycin	1	NA	2.040 (0.038-108.947)	NA	NA	NA	NA	NA	2.040 (0.038-108.947)	NA	NA
Azithromycin vs doxycycline	1	NA	2.872 (0.730-11.292)	NA	NA	0.662 (0.011-38.762)	NA	NA	2.474 (0.676-9.056)	NA	.50
Azithromycin vs minocycline	1	NA	0.276 (0.011-6.992)	NA	NA	1.197 (0.071-20.205)	NA	NA	0.634 (0.075-5.321)	NA	.50
Chloramphenicol vs clarithromycin	1	NA	1.560 (0.029-83.799)	NA	NA	NA	NA	NA	1.560 (0.029–83.799)	NA	NA
Chloramphenicol vs doxycycline	0	NA	NA	NA	NA	1.892 (0.029-122.549)	NA	NA	1.892 (0.029-122.549)	NA	NA
Chloramphenicol vs minocycline	0	NA	NA	NA	NA	0.484 (0.005-43.573)	NA	NA	0.484 (0.005-43.573)	NA	NA
Clarithromycin vs doxycycline	0	NA	NA	NA	NA	1.213 (0.018-79.601)	NA	NA	1.213 (0.018-79.601)	NA	NA
Clarithromycin vs minocycline	0	NA	NA	NA	NA	0.311 (0.003-28.276)	NA	NA	0.311 (0.003-28.276)	NA	NA
Doxycycline vs minocycline	1	NA	0.417 (0.035-4.939)	NA	NA	0.096 (0.003-3.215)	NA	NA	0.256 (0.034-1.934)	NA	.50

Abbreviations: MD, mean deviation; NA, not available; OR, odds ratio; RCT, randomized clinical trial.

^a^ The certainty of the evidence, according to Grading of Recommendations Assessment, Development, and Evaluation, was incorporated and categorized as high, moderate, low, or very low.

^b^ The results of the test for inconsistency were incorporated; *P* < .05 indicates existence of inconsistency.

^c^ Downgraded once for study limitations (risk of bias).

^d^ Downgraded once for imprecision.

^e^ Statistically significant.

To assess the defervescence time of different drugs, we analyzed separately the data of 7 treatment regimens from RCTs and data on 5 drugs from retrospective studies (Figure 2B and E). All results of the comparison are presented as the MD and 95% CIs. We compared the defervescence time of all treatment regimens with that of azithromycin (reference drug). There was no significant difference among drugs in RCTs (Figure 3B). For antibiotics in retrospective studies, the defervescence time of chloramphenicol (MD, −12.96; 95% CI, −16.49 to −9.43), clarithromycin (−23.28; 95% CI, −26.34 to −20.22), and minocycline (−18.17; 95% CI, −31.18 to −5.16) was significantly shorter than that of azithromycin (Figure 3E).

The results of pairwise comparison of all treatment regimens are presented in the Table. There was no significant difference among treatment regimens in RCTs. In retrospective studies, the defervescence time of azithromycin was longer than that of chloramphenicol (MD, 12.96; 95% CI, 9.43-16.49), clarithromycin (MD, 23.28; 95% CI, 20.22-26.34), and minocycline (MD, 18.17; 95% CI, 5.16-31.18). The defervescence time of chloramphenicol was longer than that of clarithromycin (MD, 10.32; 95% CI, 6.09-14.55). With regard to other comparisons in retrospective studies, there was no significant difference in the defervescence time among them.

All data on drug safety were collected were from RCTs only. A total of 158 patients developed an adverse reaction during or after therapy. The most frequently reported adverse reactions were vomiting, erythematous rash, gastrointestinal reaction, nausea, diarrhea, and increased serum level of alanine aminotransferase.

We assessed the safety of 7 treatment regimens (Figure 2C). A forest plot (Figure 3C) exhibited the results of comparing the safety of 7 drugs with that of azithromycin (reference drug). In addition, results of pairwise comparison of all drugs are displayed in eFigure 2 in the Supplement. There was no significant difference among the treatment regimens with regard to their safety (ie, the range of 95% CIs for all results included 1).

### Inconsistency, Ranking, and Certainty of Evidence

Results of evaluation of the inconsistency for all comparisons are presented in eFigure 2 in the Supplement and the Table. We noted a significance level of *P* > .05 for all cases, which indicated that inconsistency was not present in any comparison. All details and original data of testing inconsistency are displayed in eFigures 3, 4, 5, 6, and 7 in the Supplement.

Upon pairwise comparison of drugs, we could create a ranking on defervescence time only for those reported in retrospective studies. Clarithromycin had the shortest defervescence time and its P score was 0.8730; P scores of the other drugs were 0.6424 for minocycline, 0.5623 for doxycycline, 0.4018 for chloramphenicol, and 0.0205 for azithromycin. In addition, because there was no significant difference of efficacy, safety, and defervescence time among antibiotics reported in RCTs, we were unable to create a ranking for drugs reported in RCTs. After comparison results had been obtained, we used the GRADE system to evaluate the certainty of evidence (eFigure 2 in the Supplement; Table).

## Discussion

Scrub typhus is transmitted via mites and is a challenging problem that threatens public health. In the past, most scrub typhus cases have occurred in the “tsutsugamushi triangle,” a region covering the Russian far east in the north, Japan in the east, northern Australia in the south, and Afghanistan in the west. However, a recent report identified some cases in South America, which is an area that had no previously documented scrub typhus cases.^5^ So far, there is no available vaccine for *O tsutsugamushi* because of extensive antigenic diversity and a short duration of immune protection following immunity stimulated by naturally acquired scrub typhus infection.^12,49^ Therefore, antibiotics are the only therapy for scrub typhus. However, inappropriate treatment regimens may cause patients to become sicker or even increase the incidence of mortality.^12^ Hence, a comprehensive assessment for efficacy of different treatment regimens may be of benefit for clinicians selecting treatment for scrub typhus. So far, doxycycline, azithromycin, and chloramphenicol are common antibiotics for treating scrub typhus.^12,13^ Chloramphenicol and tetracycline are efficacious therapies, but both are contraindicated in pregnant women and children.^17^ Increasing numbers of antibiotics have been reported to be efficacious against scrub typhus.^15^

Several scholars have attempted pairwise comparisons of treatment efficacy.^13,14,15,16,17^ Nevertheless, a comprehensive ranking of various treatment regimens with regard to the efficacy and safety of drugs used against scrub typhus is lacking. Hence, we conducted a network meta-analysis to create a ranking of treatment regimens based on efficacy, safety, and the defervescence time.

Our network meta-analysis focused on 8 antibiotics involving 1211 patients by analyzing data derived from 10 RCTs and 4 retrospective studies. No specific antibiotic showed a significant advantage or disadvantage with regard to efficacy or safety. However, data derived from retrospective studies indicated that clarithromycin alleviated fever more quickly (Figure 3F). Subsequently, we tested whether inconsistency was present in our network meta-analysis. Test results suggested that there was no inconsistency because the *P* value was >.05 in all cases.

Based on the results of the study, it appears that analysis does not support the wide use of doxycycline, azithromycin, tetracycline, and chloramphenicol for treating scrub typhus. So far, these 4 antibiotics do not display better efficacy or safety than other antibiotics, which implies that these 4 antibiotics might not be the most effective therapies for treating scrub typhus and we may still need to develop more-effective treatment regimens. Because the most common manifestation of scrub typhus is fever, we also assessed the defervescence time of different antibiotics. The defervescence time of azithromycin, chloramphenicol, and doxycycline was significantly longer than that of clarithromycin and minocycline, which suggests a disadvantage of azithromycin, chloramphenicol, and doxycycline in alleviating fever and also implies that these 3 antibiotics might not be suitable for wide use for treating scrub typhus.

### Limitations

Our study had 4 main limitations. First, the total number of patients involved in this network meta-analysis was relatively small, which led to wide 95% CIs. Hence, the advantages or disadvantages of a certain drug may have been obscured. Second, some RCTs had a low level of assessment of the risk of bias, which implied that the quality of these RCTs was relatively low and led to high uncertainty of evidence. Third, only 4 retrospective studies were included in our analysis, which may lead to limited results for these studies. In addition, only 12 patients received clarithromycin. Thus, although clarithromycin showed the best results for alleviating fever, we do not think that it definitely is the first choice for fever clearance. In addition, we did not focus on therapy duration or route of administration of antibiotics owing to insufficient data. Overall, a large number of studies of high quality will need to be available to obtain more reliable results.

## Conclusions

The network meta-analysis described herein provides a reference for clinical decision-making. No treatment regimen reported in this network meta-analysis showed a significant advantage or disadvantage with regard to efficacy or safety. However, clarithromycin might be the best antibiotic for alleviating fever in people with scrub typhus.

## Notes

Supplement. **eTable 1.** All Commands Used in R and Stata **eTable 2.** Data Used for Analyzing Efficacy of Drugs **eTable 3.** Data Used for Analyzing Safety of Drugs **eTable 4.** Data Used for Analyzing the Defervescence Time of Drugs **eFigure 1.** Assessment of Risk of Bias **eFigure 2.** Pairwise Comparisons for the Efficacy and Safety of 8 Antibiotics in RCTs **eFigure 3.** Inconsistency Test of Efficacy in RCTs **eFigure 4.** Inconsistency Test of Defervescence Time in RCTs **eFigure 5.** Inconsistency Test of Safety in RCTs **eFigure 6.** Inconsistency Test of Efficacy in Retrospective Studies **eFigure 7.** Inconsistency Test of Defervescence Time in Retrospective Studies **eReferences**
