# Supplementary material for: Efficacy and Safety of Antibiotics for Treatment of Scrub Typhus: A Network Meta-analysis
Source: JAMA Netw Open. 2020 Aug 28;3(8):e2014487. doi: 10.1001/jamanetworkopen.2020.14487 (PMC7455851; doi:10.1001/jamanetworkopen.2020.14487)
Supplement: Supplement. — eTable 1. All Commands Used in R and Stata eTable 2. Data Used for Analyzing Efficacy of Drugs eTable 3. Data Used for Analyzing Safety of Drugs eTable 4. Data Used for Analyzing the Defervescence Time of Drugs eFigure 1. Assessment of Risk of Bias eFigure 2. Pairwise Comparisons for the Efficacy and Safety of 8 Antibiotics in RCTs eFigure 3. Inconsistency Test of Efficacy in RCTs eFigure 4. Inconsistency Test of Defervescence Time in RCTs eFigure 5. Inconsistency Test of Safety in RCTs eFigure 6. Inconsistency Test of Efficacy in Retrospective Studies eFigure 7. Inconsistency Test of Defervescence Time in Retrospective Studies eReferences [file jamanetwopen-3-e2014487-s001.pdf]

Supplementary Online Content

Yang J, Luo L, Chen T, et al. Efficacy and safety of antibiotics for treatment of scrub typhus: a network meta-analysis. *JAMA Netw Open*. 2020;3(8):e2014487. doi:10.1001/jamanetworkopen.2020.14487

eTable 1. All Commands Used in R and Stata

eTable 2. Data Used for Analyzing Efficacy of Drugs

eTable 3. Data Used for Analyzing Safety of Drugs

eTable 4. Data Used for Analyzing the Defervescence Time of Drugs

eFigure 1. Assessment of Risk of Bias

eFigure 2. Pairwise Comparisons for the Efficacy and Safety of 8 Antibiotics in RCTs

eFigure 3. Inconsistency Test of Efficacy in RCTs

eFigure 4. Inconsistency Test of Defervescence Time in RCTs

eFigure 5. Inconsistency Test of Safety in RCTs

eFigure 6. Inconsistency Test of Efficacy in Retrospective Studies

eFigure 7. Inconsistency Test of Defervescence Time in Retrospective Studies

eReferences

This supplementary material has been provided by the authors to give readers additional information about their work.

© 2020 Yang J et al. *JAMA Network Open.*

eTable 1: All commands used in R and STATA

Software	Package	Commands
R	Metafor	linedata<-read.csv(file=" ")
		metadata<-escalc(m1i=y1, sd1i=sd1, n1i=n1, m2i=y2, sd2i=sd2, n2i=n2, measure=" ", data=linedata)
		metadata<-transform(metadata, se=sqrt(metadata$vi))
	Netmeta	net1<-netmeta(TE=metadata$yi, seTE=metadata$se, treat1=metadata$Treatment1, treat2=metadata$Treatment2, sm=" ", studlab=metadata$Study, comb.fixed=FALSE)
		summary(net1)
		forest(net1)
		netleague(net1,digits=2)
		print(netsplit(net1),digits=2,ci=TRUE,comb.fixed=FALSE)
		netrank(net1,small.value=" ")
STATA	N/A	Networkplot t1 t2

© 2020 Yang J et al. *JAMA Network Open.*

eTable 2: Data used for analyzing efficacy of drugs

Study	Study design	Gender	Age	Length of follow-up	Intervene 1		Intervene 2		Intervene 3	
					Drug	Sample size	Drug	Sample size	Drug	Sample size
Thomas W. Sheehy, 1973[1]	RCT	Unclear	Unclear	Unclear	Chloramphenicol	30	Tetracycline	30	N/A	N/A
G. W. Brown, 1978[2]	RCT	Male & Female	Adult	14 days	Doxycycline	31	Tetracycline	24	N/A	N/A
Sakti Ranjan Paul, 1987[3]	RCT	Male & Female	Infant & adult	Unclear	Chloramphenicol	3	Tetracycline (high dosage)	16	Tetracycline	21
Jae-Hoon Song, 1995[4]	RCT	Male & Female	Adult	4 weeks	Doxycycline	66	Tetracycline	50	N/A	N/A
George Watt, 2000[5]	RCT	Male & Female	Adult	Unclear	Doxycycline	N/A	Rifampin	N/A	Rifampin (high dosage)	N/A
Yeon-Sook Kim, 2004[6]	RCT	Male & Female	Adult	2 weeks	Azithromycin	47	Doxycycline	46	N/A	N/A
Dong-Min Kim, 2007[7]	RCT	Male & Female	Adult	Unclear	Telithromycin	47	Doxycycline	45	N/A	N/A
Kriangsak Phimda, 2007[8]	RCT	Male & Female	Adult and teenager	2 weeks	Doxycycline	27	Azithromycin	29	N/A	N/A
Chen-Chi Tsai, 2011[9]	Retrospective	Male & Female	Adult	Unclear	Minocycline	25	Doxycycline	22	N/A	N/A
Mi-Ok Jang, 2014[10]	Retrospective	Male & Female	Adult	Unclear	Doxycycline	73	Azithromycin	73	N/A	N/A
Chulapong Chanta, 2015[11]	RCT	Male & Female	Children	Unclear	Azithromycin	29	Doxycycline	9	Chloramphenicol	19
Minxing Zhao, 2016[12]	Retrospective	Male & Female	Adult	30 days	Minocycline	40	Azithromycin	34	N/A	N/A
Min Lee, 2017[13]	Retrospective	Male & Female	Children	Unclear	Chloramphenicol	19	Azithromycin	25	Clarithromycin	12
Yun Sung Kim, 2018[14]	RCT	Male & Female	Adult	30 days	Doxycycline	121	Rifampin	119	N/A	N/A

© 2020 Yang J et al. *JAMA Network Open.*

eTable 3: Data used for analyzing safety of drugs

Study	Study design	Gender	Age	Length of follow-up	Intervene 1		Intervene 2		Intervene 3	
					Drug	Sample size	Drug	Sample size	Drug	Sample size
Thomas W. Sheehy, 1973[1]	RCT	Unclear	Unclear	Unclear	N/A	N/A	N/A	N/A	N/A	N/A
G. W. Brown, 1978[2]	RCT	Male & Female	Adult	14 days	Doxycycline	35	Tetracycline	30	N/A	N/A
Sakti Ranjan Paul, 1987[3]	RCT	Male & Female	Infant & adult	Unclear	Chloramphenicol	3	Tetracycline	37	Tetracycline	21
Jae-Hoon Song, 1995[4]	RCT	Male & Female	Adult	4 weeks	Doxycycline	66	Tetracycline	50	N/A	N/A
George Watt, 2000[5]	RCT	Male & Female	Adult	Unclear	Doxycycline	40	Rifampin	38	Rifampin (high dosage)	37
Yeon-Sook Kim, 2004[6]	RCT	Male & Female	Adult	2 weeks	Azithromycin	47	Doxycycline	46	N/A	N/A
Dong-Min Kim, 2007[7]	RCT	Male & Female	Adult	Unclear	Telithromycin	47	Doxycycline	45	N/A	N/A
Kriangsak Phimda, 2007[8]	RCT	Male & Female	Adult and teenager	2 weeks	N/A	N/A	N/A	N/A	N/A	N/A
Chen-Chi Tsai, 2011[9]	Retrospective	Male & Female	Adult	Unclear	N/A	N/A	N/A	N/A	N/A	N/A
Mi-Ok Jang, 2014[10]	Retrospective	Male & Female	Adult	Unclear	N/A	N/A	N/A	N/A	N/A	N/A
Chulapong Chanta, 2015[11]	RCT	Male & Female	Children	Unclear	Azithromycin	29	Doxycycline	9	Chloramphenicol	19
Minxing Zhao, 2016[12]	Retrospective	Male & Female	Adult	30 days	Minocycline	40	Azithromycin	34	N/A	N/A
Min Lee, 2017[13]	Retrospective	Male & Female	Children	Unclear	Chloramphenicol	19	Azithromycin	25	Clarithromycin	12
Yun Sung Kim, 2018[14]	RCT	Male & Female	Adult	30 days	Doxycycline	83	Rifampin	75	N/A	N/A

© 2020 Yang J et al. *JAMA Network Open.*

eTable 4: Data used for analyzing the defervescence time of drugs

Study	Intervene 1				Intervene 2				Intervene 3			
	Drug	Mean	SD	Sample size	Drug	Mean	SD	Sample size	Drug	Mean	SD	Sample size
Thomas W. Sheehy, 1973[1]	Chloramphenicol	34.7	19.5	30	Tetracycline	27.8	13.5	30	N/A	N/A	N/A	N/A
G. W. Brown, 1978[2]	N/A	N/A	N/A	N/A	N/A	N/A	N/A	N/A	N/A	N/A	N/A	N/A
Sakti Ranjan Paul, 1987[3]	N/A	N/A	N/A	N/A	N/A	N/A	N/A	N/A	N/A	N/A	N/A	N/A
Jae-Hoon Song, 1995[4]	Doxycycline	34	26.5	66	Tetracycline	37	26.6	50	N/A	N/A	N/A	N/A
George Watt, 2000[5]	Doxycycline	54.67	28.84	28	Rifampin	32.7	23.66	26	Rifampin (high dosage)	28.21	22.47	24
Yeon-Sook Kim, 2004[6]	Azithromycin	30.76	31.21	47	Doxycycline	43.02	47.83	46	N/A	N/A	N/A	N/A
Dong-Min Kim, 2007[7]	Telithromycin	20.45	12.9	47	Doxycycline	22.6	21.44	45	N/A	N/A	N/A	N/A
Kriangsak Phimda, 2007[8]	Doxycycline	54.99	30.36	27	Azithromycin	60.75	31.33	30	N/A	N/A	N/A	N/A
Chen-Chi Tsai, 2011[9]	Minocycline	17.48	9.36	40	Azithromycin	35.65	37.73	34	N/A	N/A	N/A	N/A
Mi-Ok Jang, 2014[10]	N/A	N/A	N/A	N/A	N/A	N/A	N/A	N/A	N/A	N/A	N/A	N/A
Chulapong Chanta, 2015[11]	Azithromycin	54.23	60.07	29	Doxycycline	48.69	63.7	9	Chloramphenicol	39.25	34.26	19
Minxing Zhao, 2016[12]	Minocycline	17.48	9.36	40	Azithromycin	35.65	37.73	34	N/A	N/A	N/A	N/A
Min Lee, 2017[13]	Chloramphenicol	30.24	7.2	19	Azithromycin	43.2	3.6	25	Clarithromycin	19.92	4.8	12
Yun Sung Kim, 2018[14]	Doxycycline	26.44	18.12	121	Rifampin	21.31	15.1	119	N/A	N/A	N/A	N/A

© 2020 Yang J et al. *JAMA Network Open.*

eFigure 1: Assessment of risk of bias

	Thomas W. Sheehy, 1973	G. W. Brown, 1978	Sakti Ranjan Paul, 1987	Jae-Hoon Song, 1995	George Watt, 2000	Yeon-Sook Kim, 2004	Dong-Min Kim, 2007	Kriangsak Phimda, 2007	Chulapong Chanta, 2015	Yun Sung Kim, 2018
**Random sequence generation (selection bias)**	+	+	?	+	?	+	+	+	+	+
**Allocation concealment (selection bias)**	?	?	–	–	?	–	–	+	?	–
**Blinding of participants and personnel (performance bias)**	?	?	–	–	–	–	–	–	–	–
**Blinding of outcome assessment (detection bias)**	?	?	?	?	?	?	?	?	?	?
**Incomplete outcome data (attrition bias)**	+	+	+	+	+	+	+	+	+	+
**Selective reporting(reporting bias)**	+	+	+	+	+	+	+	+	+	+
**Other bias**	+	+	–	+	–	+	+	+	–	+

Summary of the assessment of the risk of bias of RCTs. +, low risk of bias; –, high risk of bias; ?, unclear risk of bias.

© 2020 Yang J et al. *JAMA Network Open.*

eFigure 2.. Pairwise Comparisons for the Efficacy and Safety of 8 Antibiotics in RCTs

Efficacy (response rate) Comparison Safety (adverse reactions rate)

**Azithromycin**	1.441<br>[0.053-38.462]<br>P-value=0.3663<br>⊕⊕○○†*	1.004<br>[0.120-8.403]<br>P-value=0.3355<br>⊕⊕○○†*	0.617<br>[0.037-10.204]<br>⊕⊕○○†*	0.952<br>[0.038-23.810]<br>⊕⊕○○†*	0.543<br>[0.018-16.129]<br>⊕○○○†¶	0.714<br>[0.043-11.765]<br>⊕⊕○○†*	N/A
0.582<br>[0.124-2.737]<br>P-value=0.5027<br>⊕⊕○○†*	**Chloramphenicol**	0.697<br>[0.028-17.241]<br>P-value=0.5336<br>⊕⊕○○†*	0.429<br>[0.011-17.241]<br>⊕○○○†¶	0.661<br>[0.012-37.037]<br>⊕○○○†¶	0.377<br>[0.006-23.810]<br>⊕○○○†¶	0.496<br>[0.018-0.496]<br>P-value=0.3355<br>⊕⊕○○†*	N/A
1.200<br>[0.317-4.544]<br>P-value=0.5663<br>⊕⊕○○†*	2.061<br>[0.409-10.392]<br>P-value=0.8202<br>⊕⊕○○†*	**Doxycycline**	0.615<br>[0.098-3.846]<br>⊕⊕○○†*	0.949<br>[0.084-10.753]<br>P-value=0.5763<br>⊕⊕○○†*	0.541<br>[0.037-7.576]	0.711<br>[0.096-5.263]<br>P-value=0.3355<br>⊕⊕○○†*	N/A
0.403<br>[0.012-13.039]<br>⊕⊕○○†*	0.693<br>[0.019-25.234]<br>⊕○○○†¶	0.336<br>[0.014-8.334]<br>⊕⊕○○†*	**Rifampin**	1.543<br>[0.134-17.857]<br>P-value=0.5763<br>⊕⊕○○†*	0.880<br>[0.035-21.739]<br>⊕○○○†¶	1.156<br>[0.077-17.544]<br>⊕⊕○○†*	N/A
N/A	N/A	N/A	N/A	**Rifampin (high dosage)**	0.570<br>[0.016-20.408]<br>⊕○○○†¶	0.750<br>[0.032-17.544]<br>⊕⊕○○†*	N/A
1.150<br>[0.018-73.645]<br>⊕○○○†¶	1.975<br>[0.028-139.827]<br>⊕○○○†¶	0.958<br>[0.019-49.301]	2.850<br>[0.018-459.654]<br>⊕○○○†¶	N/A	**Telithromycin**	1.314<br>[0.048-35.714]<br>⊕○○○†¶	N/A
0.229<br>[0.036-1.437]<br>⊕⊕○○†*	0.392<br>[0.096-1.611]<br>P-value=0.5663<br>⊕⊕○○†*	0.190<br>[0.034-1.070]<br>P-value=0.5663<br>⊕⊕○○†*	0.566<br>[0.015-21.690]<br>⊕⊕○○†*	N/A	0.199<br>[0.003-14.685]<br>⊕○○○†¶	**Tetracycline**	N/A
0.197<br>[0.004-8.832]<br>⊕○○○†¶	0.337<br>[0.010-11.775]<br>P-value=0.6522<br>⊕⊕○○†*	0.164<br>[0.004-7.256]<br>⊕○○○†¶	0.487<br>[0.003-70.023]<br>⊕○○○†¶	N/A	0.171<br>[0.001-40.529]<br>⊕○○○†¶	0.860<br>[0.025-29.538]<br>P-value=0.6522<br>⊕⊕○○†*	**Tetracycline (high dosage)**

Drugs are reported in alphabetical order. Data are ORs (95% CI) in the column-defining treatment compared with the row-defining treatment. For efficacy, ORs >1 favor the column-defining treatment (ie, the first in alphabetical order). For safety, ORs >1 favor the first drug in alphabetical order. To obtain ORs for comparisons in the opposite direction, reciprocals should be taken. The certainty of the evidence (according to GRADE) is incorporated in this figure and categorized as high (⊕⊕⊕⊕), moderate (⊕⊕⊕O), low (⊕⊕OO), or very low (⊕OOO).

†Downgraded once for study limitations (risk of bias).

Abbreviation: OR, odds ratio.

*Downgraded once for imprecision.

¶Downgraded twice for severe imprecision. The results of the test for inconsistency was incorporated in this figure. *P* < .05 indicates existence inconsistency.

© 2020 Yang J et al. *JAMA Network Open.*

eFigure 3: Inconsistency test of efficacy in RCTs

Back-calculation method to split direct and indirect evidence

Random effects model:

comparison	k	prop	nma	95%-CI	direct	95%-CI	indir.	95%-CI	RoR	95%-CI	z	p-value
Azithromycin:Chloramphenicol	1	0.81	0.58	[0.12; 2.74]	0.45	[0.08; 2.52]	1.74	[0.05; 61.25]	0.26	[0.00; 13.48]	-0.67	0.5027
Azithromycin:Doxycycline	3	0.91	1.20	[0.32; 4.54]	1.36	[0.34; 5.50]	0.35	[0.00; 28.13]	3.83	[0.04; 377.80]	0.57	0.5663
Azithromycin:Rifampin	0	0	0.40	[0.01; 13.04]	.	.	0.40	[0.01; 13.04]	.	.	.	.
Azithromycin:Telithromycin	0	0	1.15	[0.02; 73.64]	.	.	1.15	[0.02; 73.64]	.	.	.	.
Azithromycin:Tetracycline	0	0	0.23	[0.04; 1.44]	.	.	0.23	[0.04; 1.44]	.	.	.	.
Azithromycin:Tetracycline (high dosage)	0	0	0.20	[0.00; 8.83]	.	.	0.20	[0.00; 8.83]	.	.	.	.
Chloramphenicol:Doxycycline	1	0.57	2.06	[0.41; 10.39]	2.43	[0.28; 20.82]	1.66	[0.14; 19.43]	1.46	[0.06; 38.22]	0.23	0.8202
Chloramphenicol:Rifampin	0	0	0.69	[0.02; 25.23]	.	.	0.69	[0.02; 25.23]	.	.	.	.
Chloramphenicol:Telithromycin	0	0	1.97	[0.03; 139.83]	.	.	1.97	[0.03; 139.83]	.	.	.	.
Chloramphenicol:Tetracycline	2	0.79	0.39	[0.10; 1.61]	0.32	[0.06; 1.55]	0.87	[0.04; 18.85]	0.36	[0.01; 11.56]	-0.57	0.5663
Chloramphenicol:Tetracycline (high dosage)	1	0.76	0.34	[0.01; 11.78]	0.21	[0.00; 12.62]	1.42	[0.00; 1879.35]	0.15	[0.00; 582.32]	-0.45	0.6522
Doxycycline:Rifampin	1	1.00	0.34	[0.01; 8.33]	0.34	[0.01; 8.33]	.	.	.	.	.	.
Doxycycline:Telithromycin	1	1.00	0.96	[0.02; 49.30]	0.96	[0.02; 49.30]	.	.	.	.	.	.
Doxycycline:Tetracycline	2	0.53	0.19	[0.03; 1.07]	0.31	[0.03; 3.24]	0.11	[0.01; 1.39]	2.75	[0.09; 87.57]	0.57	0.5663
Doxycycline:Tetracycline (high dosage)	0	0	0.16	[0.00; 7.26]	.	.	0.16	[0.00; 7.26]	.	.	.	.
Rifampin:Telithromycin	0	0	2.85	[0.02; 459.65]	.	.	2.85	[0.02; 459.65]	.	.	.	.
Rifampin:Tetracycline	0	0	0.57	[0.01; 21.69]	.	.	0.57	[0.01; 21.69]	.	.	.	.
Rifampin:Tetracycline (high dosage)	0	0	0.49	[0.00; 70.02]	.	.	0.49	[0.00; 70.02]	.	.	.	.
Telithromycin:Tetracycline	0	0	0.20	[0.00; 14.68]	.	.	0.20	[0.00; 14.68]	.	.	.	.
Telithromycin:Tetracycline (high dosage)	0	0	0.17	[0.00; 40.53]	.	.	0.17	[0.00; 40.53]	.	.	.	.
Tetracycline:Tetracycline (high dosage)	1	0.79	0.86	[0.03; 29.54]	1.30	[0.02; 69.18]	0.18	[0.00; 414.94]	7.44	[0.00; 45845.71]	0.45	0.6522

Legend:
- comparison - Treatment comparison
- k - Number of studies providing direct evidence
- prop - Direct evidence proportion
- nma - Estimated treatment effect (OR) in network meta-analysis
- direct - Estimated treatment effect (OR) derived from direct evidence
- indir. - Estimated treatment effect (OR) derived from indirect evidence
- RoR - Ratio of Ratios (direct versus indirect)
- z - z-value of test for disagreement (direct versus indirect)
- p-value - p-value of test for disagreement (direct versus indirect)

© 2020 Yang J et al. *JAMA Network Open.*

eFigure 4: Inconsistency test of defervescence time in RCTs

Back-calculation method to split direct and indirect evidence

Random effects model:

comparison	k	prop	nma	95%-CI	direct	95%-CI	indir.	95%-CI	Diff	95%-CI	z	p-value
Azithromycin:Chloramphenicol	1	0.52	2.11	[-21.62; 25.84]	14.98	[-18.05; 48.01]	-11.62	[-45.75; 22.50]	26.60	[-20.88; 74.09]	1.10	0.2722
Azithromycin:Doxycycline	3	0.92	0.46	[-15.73; 16.65]	-2.20	[-19.06; 14.67]	31.59	[-26.15; 89.33]	-33.78	[-93.94; 26.37]	-1.10	0.2710
Azithromycin:Rifampin	0	0	12.45	[-9.81; 34.70]	.	.	12.45	[-9.81; 34.70]	.	.	.	.
Azithromycin:Rifampin (high dosage)	0	0	21.66	[-5.52; 48.83]	.	.	21.66	[-5.52; 48.83]	.	.	.	.
Azithromycin:Telithromycin	0	0	2.61	[-23.67; 28.89]	.	.	2.61	[-23.67; 28.89]	.	.	.	.
Azithromycin:Tetracycline	0	0	3.38	[-19.47; 26.23]	.	.	3.38	[-19.47; 26.23]	.	.	.	.
Chloramphenicol:Doxycycline	1	0.21	-1.65	[-23.91; 20.61]	-9.44	[-57.86; 38.98]	0.44	[-24.63; 25.51]	-9.88	[-64.41; 44.65]	-0.36	0.7226
Chloramphenicol:Rifampin	0	0	10.34	[-16.66; 37.34]	.	.	10.34	[-16.66; 37.34]	.	.	.	.
Chloramphenicol:Rifampin (high dosage)	0	0	19.55	[-11.63; 50.72]	.	.	19.55	[-11.63; 50.72]	.	.	.	.
Chloramphenicol:Telithromycin	0	0	0.50	[-29.90; 30.90]	.	.	0.50	[-29.90; 30.90]	.	.	.	.
Chloramphenicol:Tetracycline	1	0.78	1.27	[-17.36; 19.90]	6.90	[-14.26; 28.06]	-18.18	[-57.52; 21.15]	25.08	[-19.58; 69.75]	1.10	0.2710
Doxycycline:Rifampin	2	1.00	11.99	[-3.28; 27.26]	11.99	[-3.28; 27.26]	.	.	.	.	.	.
Doxycycline:Rifampin (high dosage)	1	0.83	21.20	[-0.63; 43.02]	26.46	[2.57; 50.35]	-5.36	[-59.02; 48.29]	31.82	[-26.91; 90.55]	1.06	0.2882
Doxycycline:Telithromycin	1	1.00	2.15	[-18.55; 22.85]	2.15	[-18.55; 22.85]	.	.	.	.	.	.
Doxycycline:Tetracycline	1	0.76	2.92	[-16.05; 21.89]	-3.00	[-24.70; 18.70]	22.08	[-16.95; 61.12]	-25.08	[-69.75; 19.58]	-1.10	0.2710
Rifampin:Rifampin (high dosage)	1	0.86	9.21	[-12.32; 30.73]	4.49	[-18.73; 27.71]	38.05	[-19.37; 95.48]	-33.56	[-95.50; 28.38]	-1.06	0.2882
Rifampin:Telithromycin	0	0	-9.84	[-35.56; 15.88]	.	.	-9.84	[-35.56; 15.88]	.	.	.	.
Rifampin:Tetracycline	0	0	-9.07	[-33.42; 15.28]	.	.	-9.07	[-33.42; 15.28]	.	.	.	.
Rifampin (high dosage):Telithromycin	0	0	-19.05	[-49.12; 11.03]	.	.	-19.05	[-49.12; 11.03]	.	.	.	.
Rifampin (high dosage):Tetracycline	0	0	-18.27	[-47.19; 10.64]	.	.	-18.27	[-47.19; 10.64]	.	.	.	.
Telithromycin:Tetracycline	0	0	0.77	[-27.30; 28.85]	.	.	0.77	[-27.30; 28.85]	.	.	.	.

Legend:
comparison - Treatment comparison
k - Number of studies providing direct evidence
prop - Direct evidence proportion
nma - Estimated treatment effect (MD) in network meta-analysis
direct - Estimated treatment effect (MD) derived from direct evidence
indir. - Estimated treatment effect (MD) derived from indirect evidence
Diff - Difference between direct and indirect treatment estimates
z - z-value of test for disagreement (direct versus indirect)
p-value - p-value of test for disagreement (direct versus indirect)

© 2020 Yang J et al. *JAMA Network Open.*

eFigure 5: Inconsistency test of safety in RCTs

Back-calculation method to split direct and indirect evidence

Random effects model:

comparison	k	prop	nma	95%-CI	direct	95%-CI	indir.	95%-CI	RoR	95%-CI	z	p-value
Azithromycin:Chloramphenicol	1	0.66	0.69	[0.03; 18.85]	2.05	[0.04; 118.15]	0.08	[0.00; 24.27]	25.04	[0.02; 27080.88]	0.90	0.3663
Azithromycin:Doxycycline	2	0.95	1.00	[0.12; 8.33]	0.79	[0.09; 6.95]	105.25	[0.01; 1751868.38]	0.01	[0.00; 158.58]	-0.96	0.3355
Azithromycin:Rifampin	0	0	1.62	[0.10; 26.78]	.	.	1.62	[0.10; 26.78]	.	.	.	.
Azithromycin:Rifampin (high dosage)	0	0	1.05	[0.04; 26.36]	.	.	1.05	[0.04; 26.36]	.	.	.	.
Azithromycin:Telithromycin	0	0	1.84	[0.06; 54.43]	.	.	1.84	[0.06; 54.43]	.	.	.	.
Azithromycin:Tetracycline	0	0	1.40	[0.08; 23.19]	.	.	1.40	[0.08; 23.19]	.	.	.	.
Chloramphenicol:Doxycycline	1	0.47	1.43	[0.06; 35.31]	0.49	[0.00; 52.03]	3.74	[0.05; 305.64]	0.13	[0.00; 79.85]	-0.62	0.5336
Chloramphenicol:Rifampin	0	0	2.33	[0.06; 93.47]	.	.	2.33	[0.06; 93.47]	.	.	.	.
Chloramphenicol:Rifampin (high dosage)	0	0	1.51	[0.03; 84.02]	.	.	1.51	[0.03; 84.02]	.	.	.	.
Chloramphenicol:Telithromycin	0	0	2.65	[0.04; 168.13]	.	.	2.65	[0.04; 168.13]	.	.	.	.
Chloramphenicol:Tetracycline	1	0.48	2.02	[0.07; 54.44]	10.71	[0.09; 1219.35]	0.42	[0.00; 41.34]	25.54	[0.03; 18678.22]	0.96	0.3355
Doxycycline:Rifampin	2	1.00	1.63	[0.26; 10.16]	1.63	[0.26; 10.16]	.	.	.	.	.	.
Doxycycline:Rifampin (high dosage)	1	0.88	1.05	[0.09; 11.91]	1.36	[0.10; 18.15]	0.17	[0.00; 169.55]	8.24	[0.01; 13471.42]	0.56	0.5763
Doxycycline:Telithromycin	1	1.00	1.85	[0.13; 25.84]	1.85	[0.13; 25.84]	.	.	.	.	.	.
Doxycycline:Tetracycline	2	0.90	1.41	[0.19; 10.42]	1.01	[0.12; 8.35]	25.75	[0.05; 13289.76]	0.04	[0.00; 28.62]	-0.96	0.3355
Rifampin:Rifampin (high dosage)	1	0.87	0.65	[0.06; 7.48]	0.49	[0.04; 6.83]	3.80	[0.00; 3004.73]	0.13	[0.00; 168.53]	-0.56	0.5763
Rifampin:Telithromycin	0	0	1.14	[0.05; 28.20]	.	.	1.14	[0.05; 28.20]	.	.	.	.
Rifampin:Tetracycline	0	0	0.86	[0.06; 13.05]	.	.	0.86	[0.06; 13.05]	.	.	.	.
Rifampin (high dosage):Telithromycin	0	0	1.75	[0.05; 63.08]	.	.	1.75	[0.05; 63.08]	.	.	.	.
Rifampin (high dosage):Tetracycline	0	0	1.33	[0.06; 30.97]	.	.	1.33	[0.06; 30.97]	.	.	.	.
Telithromycin:Tetracycline	0	0	0.76	[0.03; 20.87]	.	.	0.76	[0.03; 20.87]	.	.	.	.

Legend:
- comparison - Treatment comparison
- k - Number of studies providing direct evidence
- prop - Direct evidence proportion
- nma - Estimated treatment effect (OR) in network meta-analysis
- direct - Estimated treatment effect (OR) derived from direct evidence
- indir. - Estimated treatment effect (OR) derived from indirect evidence
- RoR - Ratio of Ratios (direct versus indirect)
- z - z-value of test for disagreement (direct versus indirect)
- p-value - p-value of test for disagreement (direct versus indirect)

© 2020 Yang J et al. *JAMA Network Open.*

eFigure 6: Inconsistency test of efficacy in retrospective studies

Back-calculation method to split direct and indirect evidence

Random effects model:

comparison	k	prop	nma	95%-CI	direct	95%-CI	indir.	95%-CI	RoR	95%-CI	z	p-value
Azithromycin:Chloramphenicol	1	1.00	1.31	[0.02; 68.87]	1.31	[0.02; 68.87]	.	.	.	.	.	.
Azithromycin:Clarithromycin	1	1.00	2.04	[0.04; 108.95]	2.04	[0.04; 108.95]	.	.	.	.	.	.
Azithromycin:Doxycycline	1	0.90	2.47	[0.68; 9.06]	2.87	[0.73; 11.29]	0.66	[0.01; 38.76]	4.34	[0.06; 318.08]	0.67	0.5029
Azithromycin:Minocycline	1	0.43	0.63	[0.08; 5.32]	0.28	[0.01; 6.99]	1.20	[0.07; 20.21]	0.23	[0.00; 16.89]	-0.67	0.5029
Chloramphenicol:Clarithromycin	1	1.00	1.56	[0.03; 83.80]	1.56	[0.03; 83.80]	.	.	.	.	.	.
Chloramphenicol:Doxycycline	0	0	1.89	[0.03; 122.55]	.	.	1.89	[0.03; 122.55]	.	.	.	.
Chloramphenicol:Minocycline	0	0	0.48	[0.01; 43.57]	.	.	0.48	[0.01; 43.57]	.	.	.	.
Clarithromycin:Doxycycline	0	0	1.21	[0.02; 79.60]	.	.	1.21	[0.02; 79.60]	.	.	.	.
Clarithromycin:Minocycline	0	0	0.31	[0.00; 28.28]	.	.	0.31	[0.00; 28.28]	.	.	.	.
Doxycycline:Minocycline	1	0.67	0.26	[0.03; 1.93]	0.42	[0.04; 4.94]	0.10	[0.00; 3.21]	4.34	[0.06; 318.08]	0.67	0.5029

Legend:
- comparison - Treatment comparison
- k - Number of studies providing direct evidence
- prop - Direct evidence proportion
- nma - Estimated treatment effect (OR) in network meta-analysis
- direct - Estimated treatment effect (OR) derived from direct evidence
- indir. - Estimated treatment effect (OR) derived from indirect evidence
- RoR - Ratio of Ratios (direct versus indirect)
- z - z-value of test for disagreement (direct versus indirect)
- p-value - p-value of test for disagreement (direct versus indirect)

© 2020 Yang J et al. *JAMA Network Open.*

eFigure 7: Inconsistency test of defervescence time in retrospective studies

Back-calculation method to split direct and indirect evidence

Random effects model:

comparison	k	prop	nma	95%-CI	direct	95%-CI	indir.	95%-CI	Diff	95%-CI	z	p-value
Azithromycin:Chloramphenicol	1	1.00	12.96	[9.43; 16.49]	12.96	[9.43; 16.49]	.	.	.	.	.	.
Azithromycin:Clarithromycin	1	1.00	23.28	[20.22; 26.34]	23.28	[20.22; 26.34]	.	.	.	.	.	.
Azithromycin:Doxycycline	0	0	16.47	[-6.37; 39.31]	.	.	16.47	[-6.37; 39.31]	.	.	.	.
Azithromycin:Minocycline	1	1.00	18.17	[5.16; 31.18]	18.17	[5.16; 31.18]	.	.	.	.	.	.
Chloramphenicol:Clarithromycin	1	1.00	10.32	[6.09; 14.55]	10.32	[6.09; 14.55]	.	.	.	.	.	.
Chloramphenicol:Doxycycline	0	0	3.51	[-19.60; 26.62]	.	.	3.51	[-19.60; 26.62]	.	.	.	.
Chloramphenicol:Minocycline	0	0	5.21	[-8.27; 18.69]	.	.	5.21	[-8.27; 18.69]	.	.	.	.
Clarithromycin:Doxycycline	0	0	-6.81	[-29.85; 16.23]	.	.	-6.81	[-29.85; 16.23]	.	.	.	.
Clarithromycin:Minocycline	0	0	-5.11	[-18.47; 8.25]	.	.	-5.11	[-18.47; 8.25]	.	.	.	.
Doxycycline:Minocycline	1	1.00	1.70	[-17.07; 20.47]	1.70	[-17.07; 20.47]	.	.	.	.	.	.

Legend:
- comparison - Treatment comparison
- k - Number of studies providing direct evidence
- prop - Direct evidence proportion
- nma - Estimated treatment effect (MD) in network meta-analysis
- direct - Estimated treatment effect (MD) derived from direct evidence
- indir. - Estimated treatment effect (MD) derived from indirect evidence
- Diff - Difference between direct and indirect treatment estimates
- z - z-value of test for disagreement (direct versus indirect)
- p-value - p-value of test for disagreement (direct versus indirect)

© 2020 Yang J et al. *JAMA Network Open.*

eReferences

1. Sheehy TW, Hazlett D, Turk RE. Scrub typhus. A comparison of chloramphenicol and tetracycline in its treatment. *Arch Intern Med.* 1973;132(1):77-80.
2. Brown GW, Saunders JP, Singh S. Single dose doxycycline therapy for scrub typhus. *Transactions of the Royal Society of Tropical Medicine and Hygiene.* 1978;72(4):412-416.
3. Paul SR, Karanth S, Dickson C. Scrub typhus along the Thai-Kampuchean border: new treatment regimen. *Trop Doct.* 1987;17(3):104-107.
4. Song JH, Lee C, Chang WH, et al. Short-course doxycycline treatment versus conventional tetracycline therapy for scrub typhus: a multicenter randomized trial. *Clin Infect Dis.* 1995;21(3):506-510.
5. Watt G, Kantipong P, Jongsakul K, Watcharapichat P, Phulsuksombati D, Strickman D. Doxycycline and rifampicin for mild scrub-typhus infections in northern Thailand: a randomised trial. *Lancet.* 2000;356(9235):1057-1061.
6. Kim YS, Yun HJ, Shim SK, Koo SH, Kim SY, Kim S. A comparative trial of a single dose of azithromycin versus doxycycline for the treatment of mild scrub typhus. *Clin Infect Dis.* 2004;39(9):1329-1335.
7. Kim DM, Ki DY, Ji HL, Hyun KK, Lee SH. Controlled trial of a 5-day course of telithromycin versus doxycycline for treatment of mild to moderate scrub typhus. *Antimicrobial Agents and Chemotherapy.* 2007;51(6):2011-2015.
8. Phimda K, Hoontrakul S, Suttinont C, et al. Doxycycline versus azithromycin for treatment of leptospirosis and scrub typhus. *Antimicrob Agents Chemother.* 2007;51(9):3259-3263.
9. Tsai CC, Lay CJ, Ho YH, Wang LS, Chen LK. Intravenous minocycline versus oral doxycycline for the treatment of noncomplicated scrub typhus. *J Microbiol Immunol Infect.* 2011;44(1):33-38.
10. Jang MO, Jang HC, Kim UJ, et al. Outcome of intravenous azithromycin therapy in patients with complicated scrub typhus compared with that of doxycycline therapy using propensity-matched analysis. *Antimicrob Agents Chemother.* 2014;58(3):1488-1493.
11. Chanta C, Phloenchaiwanit P. Randomized Controlled Trial of Azithromycin versus Doxycycline or Chloramphenicol for Treatment of Uncomplicated Pediatric Scrub Typhus. *J Med Assoc Thai.* 2015;98(8):756-760.
12. Zhao M, Wang T, Yuan X, Du W, Lin M, Shen Y. Comparison of minocycline and azithromycin for the treatment of mild scrub typhus in northern China. *Int J Antimicrob Agents.* 2016;48(3):317-320.
13. Lee M, Kim J, Jo DS. Effects of clarithromycin treatment in scrub typhus in children: comparison with chloramphenicol and azithromycin. *Korean J Pediatr.* 2017;60(4):124-127.
14. Kim YS, Kim DM, Yoon NR, Jang MS, Kim CM. Effects of Rifampin and Doxycycline Treatments in Patients With Uncomplicated Scrub Typhus: An Open-Label, Randomized, Controlled Trial. *Clin Infect Dis.* 2018;67(4):600-605.

© 2020 Yang J et al. *JAMA Network Open.*
